# K121Q snp of ENPP1 gene is associated with acute rejection in kidney transplantation

**DOI:** 10.1186/1758-5996-7-S1-A11

**Published:** 2015-11-11

**Authors:** Denise Alves Sortica, Bruna Bellincanta Nicoletto, Pâmela Sachs Nique, Laura Bem Olivo, Evylyny Gomes Malaquias, Andrea Carla Bauer, Daisy Crispim, Roberto Ceratti Manfro, Luis Henrique Canani

**Affiliations:** 1 Universidade Federal do Rio Grande do Sul, Porto Alegre, Brazil

## Background

Diabetic kidney disease (DKD) is a common microvascular chronic complication affecting approximately 40% of patients with diabetes mellitus (DM). DKD is one of the major causes of kidney failure in many countries, and is associated with increased health system costs. Kidney transplantation is the treatment of choice for a significant portion of patients with end-stage kidney disease, including DM patients. In this context, acute rejection (AR) is a major post-transplant complication. The use of biomarkers as a method to prognosticate or detect early pathologic events in kidney transplantation is an attractive and needed strategy. Several studies have evaluated the relevance of genetic variants, including the K121Q polymorphism (rs1044498) in the ENPP1 gene, as predictors for the development of diabetes, DKD and, more recently, AR in kidney transplantation.

## Objective

The aim of this study was to evaluate the association of the ENPP1 K121Q polymorphism with acute kidney rejection.

## Materials and methods

We performed a retrospective cohort study in 407 white kidney transplant recipients from Southern Brazil. Demographic and clinical data were collected. The ENPP1 K121Q polymorphism was genotyped by real-time PCR using TaqMan MGB probes (Life Technologies). Cox regression analysis was used to evaluate overall survival of patients according to the presence of the 121Q allele and AR. This study was approved by the Ethics Committee of Hospital involved, and all subjects signed the informed consent.

## Results

Among patients who had AR, 22.3% were K allele carries (K/K or K/Q) and 42.9% showed the Q/Q genotype (P=0.03). After controlling for potential confounders (age, gender, HLA matching, delayed graft function, blood transfusions and number of pregnancies), the Q/Q genotype remained as an independent predictor of AR compared with the K allele (Harzard Ratio=2.19, 95% CI 1.10-4.35, P=0.025).

## Conclusion

The ENPP1 K121Q polymorphism was independently associated with AR in white kidney transplant recipients. If confirmed, this finding may represent a new genetic tool to predict AR.

